# Pepsinogen ratio and brachial-ankle pulse wave velocity: a cross-sectional study on their interrelationship in atherosclerosis

**DOI:** 10.1186/s12872-023-03618-9

**Published:** 2023-11-21

**Authors:** Yuexi Li, Xiaoqin Liu, Yuhan Luo, Qiaoli Wang

**Affiliations:** Health Management Center, Deyang People’s Hospital, No. 173, Taishan North Road, Deyang City, Sichuan Province China

**Keywords:** Pepsinogen I, Pepsinogen II, Pepsinogen ratio, Brachial-ankle pulse wave velocity, Atherosclerosis, Correlation analysis

## Abstract

**Background:**

Existing research has established the pepsinogen ratio (PGR) as a complex biomarker, not only as an independent predictor for various gastrointestinal diseases but also in its association with atherosclerotic cardiovascular diseases. However, the precise mechanism linking changes in PGR to cardiovascular pathologies remains unclear. The objective of this study is to quantitatively elucidate the association between PGR and brachial-ankle pulse wave velocity (baPWV) as an indicator of atherosclerotic progression.

**Methods:**

We conducted a cross-sectional study that analyzed clinical data from 465 patients who underwent health screenings. One-way Analysis of Variance (ANOVA) identified potential risk factors affecting baPWV. Multiple logistic regression was employed to evaluate if PGR serves as an independent risk factor for elevated baPWV after accounting for these variables. Generalized additive models and smoothed curve fitting were utilized to investigate the possibility of a nonlinear association between PGR and baPWV. When such nonlinearity was found, threshold effect analysis pinpointed the inflection point in this relationship, followed by segmented correlation analyses.

**Results:**

PGR negatively correlated with both right baPWV (RbaPWV) and left baPWV (LbaPWV) after adjusting for confounders. Smoothed curve analyses revealed nonlinear relationships, with inflection points at 22.5 for RbaPWV and 22.3 for LbaPWV. For PGR values below 22.5, a significant negative correlation with RbaPWV was observed (β = − 6.3 cm/s, *P* < 0.001). Conversely, for PGR values above 22.5, no significant linear relationship was found (*P* = 0.141). Similarly, when PGR was below 22.3, a strong negative correlation with LbaPWV was detected (β = − 7.0 cm/s, *P* < 0.001), but such correlation was absent for higher PGR levels (*P* = 0.273).

**Conclusion:**

The study reveals that PGR is associated with RbaPWV and LbaPWV in a nonlinear manner. Specifically, lower levels of PGR were linearly and inversely correlated with baPWV, but this relationship became nonlinear at higher PGR levels. These findings suggest that modulating PGR levels may offer a therapeutic strategy for managing atherosclerosis.

**Supplementary Information:**

The online version contains supplementary material available at 10.1186/s12872-023-03618-9.

## Introduction

Pepsinogen (PG) is a precursor enzyme with roles in digestion. It exists in seven distinct isoforms, categorized as PG1 through PG7, based on their electrophoretic mobility in agar gel—from fastest to slowest. Isoforms PG1 through PG5 possess common immunogenic properties and are collectively termed PGI. These isoforms are secreted primarily by the gastric acid-producing glands located in the fundus and body of the stomach [1]. On the other hand, the slow-migrating isoforms PG6 and PG7 are grouped together as PGII and are co-secreted by both gastric and duodenal glands [2, 3].

The pepsinogen ratio (PGR), which is the ratio of PGI to PGII levels, is frequently employed in clinical settings for the assessment of chronic atrophic gastritis and the risk of developing gastric cancer [4–6]. Thresholds like PGI ≤ 70 ng/ml and PGR ≤ 3 have been established as critical values for these gastrointestinal conditions [7].

Beyond its relevance to gastrointestinal diseases, PGR has increasingly been implicated in non-gastrointestinal conditions as well. For instance, elevated PGR levels have been linked to metabolic imbalances, such as increased levels of glucose, triglyceride, and uric acid [8]. Importantly, PGR has been identified as a predictive biomarker for atherosclerotic cardiovascular disease [9] and has been correlated with complications arising from hypertension [10]. These observations suggest the possibility of shared risk factors between cardiovascular and gastrointestinal diseases, potentially mediated through alterations in PGR metabolism. Despite these preliminary findings, the specific role of PGR in cardiovascular diseases remains unclear.

In recent years, pulse wave velocity has emerged as a reliable metric for gauging vascular stiffness, a characteristic feature of atherosclerotic pathologies [11]. Therefore, this study aims to elucidate the impact of PGR on the atherosclerotic process by quantifying its relationship with pulse wave velocity.

## Methods and materials

### Study population

This cross-sectional study was conducted during the first and second quarters of 2023 at the Health Management Center of the People’s Hospital of Deyang City. The facility predominantly carries out health screenings for adults, and 574 patients aged 18 and over voluntarily participated in PGR testing during the study timeframe. After applying exclusion criteria—such as recent use of antibiotics, gastric mucosal protectants, or acid suppressants (*n* = 16); history of gastrointestinal tumors (*n* = 3), liver cirrhosis (*n* = 18); or lack of atherosclerosis testing (*n* = 72) —the final study sample consisted of 465 patients. Ethical approval for the study was granted by the Ethics Committee of the People’s Hospital of Deyang City (Ethical Review Approval No. LWH-OP-006-A04-V2.0). Given that the study relied on existing clinical data, the Ethics Committee of the People’s Hospital of Deyang City waived the requirement of informed consent.

### Clinical and biochemical measurements

Patients’ venous blood samples were collected after an overnight fast of at least 8 h for comprehensive biochemical profiling. This included counts for red blood cells (RBC), white blood cells (WBC), and platelets, along with measurements of alanine transaminase (ALT), aspartate transaminase (AST), triglycerides (TG), total cholesterol (TC), low-density lipoprotein cholesterol (LDL-C), high-density lipoprotein cholesterol (HDL-C), fasting blood glucose, creatinine, uric acid, blood urea nitrogen, pepsinogen I, and pepsinogen II. The assays were conducted using a Beckman 5800 fully automated biochemical analyzer (Beckman Coulter Co., Ltd., USA) and a Sysmex XE-2100 analyzer (Sysmex Corporation, Japan).

Demographic and anthropometric data including age, gender, height, weight, Body Mass Index (BMI), blood pressure, and waist circumference were also collected. BMI, a key indicator for categorizing overweight or obesity, was calculated as weight (kg) divided by the square of height (m). PGR was defined as pepsinogen I level divided by pepsinogen II level.

Based on the PGR levels, patients were stratified into three groups. Low (PGR between 1.04 and 11.39), moderate (PGR between 11.42 and 16.33), and high (PGR between 16.35 and 49.02). Brachial-ankle pulse wave velocity (baPWV) was assessed using the Yueqi Atherosclerosis Detector (Model: VBP-9B) following 5 min of quiet rest. The left and right brachial-ankle PWV were denoted as LbaPWV and RbaPWV, respectively.

### Lifestyle factors

Participants were categorized into smoking and non-smoking groups based on their tobacco consumption habits. Participants who consumed tobacco at a rate exceeding one cigarette per day for a minimum duration of six continuous or cumulative months were classified into the “smoking” group. Those who smoked more than four cigarettes weekly, but fewer than one cigarette daily, were designated as “occasional smokers.” All remaining subjects were placed in the “non-smoking” category [12]. Alcohol consumption was similarly categorized. The sample was stratified into three groups: the “drinking” group (those consuming alcohol more than once a month), the “occasional drinking” group (those who consumed alcohol less frequently than once a month but more than once per year), and the “non-drinking” group (those consuming alcohol fewer than once a year) [13]. Physical activity levels of the study participants were assessed using the International Physical Activity Questionnaire (IPAQ) short-form to calculate the metabolic equivalents of task (METs). Patients with a METs score equal to or exceeding 3000 were categorized as engaging in “high-intensity physical activity.” Those with METs scores ranging from 600 to 3000 were classified into the “moderate-intensity physical activity” group. Patients with METs scores below 600 were grouped under “low-intensity physical activity” [14]. Note: METs is a unit used for estimating the amount of oxygen used by the body during physical activity. The IPAQ short-form is a validated instrument for gauging physical activity levels across different age groups and populations.

### Statistical analysis

Continuous variables are presented as mean ± standard deviation (SD), whereas categorical variables are reported as counts (percentages). To assess statistical differences across the low, moderate, and high PGR level groups for both continuous and categorical variables, the Kruskal-Wallis H test and the chi-square test were utilized, respectively. A One-way analysis of variance (ANOVA) was conducted to investigate correlations between all variables and both LbaPWV and RbaPWV. Subsequently, multiple logistic regression analysis was performed to elucidate the independent associations between PGR levels and LbaPWV or RbaPWV, after adjusting for potential confounding variables identified through the one-way ANOVA. To explore potential non-linear relationships between PGR and LbaPWV/RbaPWV, Generalized Additive Models (GAM) and smooth curve fitting were employed. Note that GAM are flexible models used to describe relationships between variables, allowing for non-linear associations. Threshold effect analysis was also conducted to identify the inflection points in the non-linear relationships between PGR and LbaPWV or RbaPWV. All statistical analyses were executed using SPSS (version 25.0), EmpowerStats software (www.empowerstats.com), and R software. A *p*-value less than 0.05 was deemed statistically significant.

## Results

### Baseline characteristics

This study encompassed a total of 465 patients with a mean age of 49.1 ± 9.5 years. The gender distribution was 54.2% male and 45.8% female. Table 1 outlines the baseline clinical features of the patients across different PGR level groups. No significant differences were observed in various categories including gender; hematological markers (such as white blood cell count and platelet count); anthropometric measurements (for example, height, weight, and BMI); biochemical tests (including ALT, AST, TG, and TC); and lifestyle habits (like smoking and alcohol consumption) among the different PGR level groups (*p* > 0.05). However, it was noteworthy that the moderate and high PGR level groups were characterized by a younger age, higher red blood cell count, and slower LbaPWV and RbaPWV compared to the low PGR level group (*p* < 0.05).

**Table 1:** Baseline clinical characteristics across different PGR level groups

	Low PGR Group	Middle PGR Group	High PGR Group	*P*-value
number	155	155	155	
SEX(n/%)				0.074
woman	74 (47.7%)	84 (54.2%)	94 (60.6%)	
man	81 (52.3%)	71 (45.8%)	61 (39.4%)	
Age (years, mean ± SD)	51.2 ± 9.0	48.8 ± 9.6	47.3 ± 9.4	< 0.001
RBC (*10^12/L, mean ± SD)	4.8 ± 0.5	4.9 ± 0.5	5.0 ± 0.5	0.007
WBC (*10^9/L, mean ± SD)	5.7 ± 1.3	5.9 ± 1.5	6.1 ± 1.8	0.107
Platelets (*10^9/L, mean ± SD)	185.6 ± 56.7	194.8 ± 58.6	194.3 ± 61.0	0.299
Height (cm, mean ± SD)	161.2 ± 8.4	161.5 ± 8.1	163.0 ± 8.7	0.118
Weight (Kg, mean ± SD)	63.3 ± 11.9	63.3 ± 10.9	63.8 ± 11.3	0.892
BMI (kg/m2, mean ± SD)	24.2 ± 3.4	24.2 ± 3.3	23.9 ± 2.9	0.561
Waist (cm, mean ± SD)	83.2 ± 11.0	83.1 ± 9.9	83.0 ± 9.5	0.985
SBP (mmHg, mean ± SD)	126.9 ± 17.9	123.5 ± 15.5	123.2 ± 15.7	0.093
DBP (mmHg, mean ± SD)	75.4 ± 11.7	75.2 ± 10.0	74.8 ± 10.8	0.854
ALT (U/L, mean ± SD)	23.3 ± 15.9	25.6 ± 22.3	28.1 ± 24.5	0.129
AST (U/L, mean ± SD)	23.9 ± 8.8	24.8 ± 16.5	26.1 ± 16.7	0.418
TG (mmol/L, mean ± SD)	163.4 ± 179.5	181.0 ± 181.2	159.2 ± 114.8	0.452
TC (mmol/L, mean ± SD)	196.2 ± 37.8	194.0 ± 39.2	189.1 ± 31.1	0.208
HDL-C (mmol/L, mean ± SD)	65.2 ± 18.5	67.1 ± 17.1	69.3 ± 15.1	0.107
LDL-C (mmol/L, mean ± SD)	110.1 ± 23.9	112.1 ± 30.9	108.4 ± 26.5	0.500
Fasting blood glucose (mmol/L, mean ± SD)	5.2 ± 0.9	5.2 ± 0.9	5.3 ± 1.2	0.625
Urea nitrogen (mmol/L, mean ± SD)	5.3 ± 1.3	5.3 ± 1.3	5.3 ± 1.2	0.974
Uric acid (umol /L, mean ± SD)	341.5 ± 89.1	349.3 ± 92.7	357.0 ± 94.6	0.335
Creatinine (umol /L, mean ± SD)	66.5 ± 15.1	69.1 ± 14.3	70.5 ± 14.1	0.052
Pepsinogen ratio (, mean ± SD)	8.3 ± 2.2	13.7 ± 1.4	20.8 ± 5.0	< 0.001
right pulse wave propagation speed (cm/s, mean ± SD)	1635.4 ± 226.3	1549.8 ± 232.7	1506.4 ± 229.0	< 0.001
Left pulse wave propagation speed (cm/s, mean ± SD)	1664.9 ± 246.3	1573.5 ± 246.2	1527.0 ± 243.6	< 0.001
Smoking(n/%)				0.144
Nonsmokers	118 (76.6%)	112 (72.3%)	117 (75.5%)	
Occasional smoking	10 (6.5%)	12 (7.7%)	3 (1.9%)	
Smokers	26 (16.9%)	31 (20.0%)	35 (22.6%)	
Drinking(n/%)				0.380
Nondrinkers	81 (52.6%)	86 (55.5%)	81 (52.3%)	
Occasional alcohol consumption	47 (30.5%)	38 (24.5%)	36 (23.2%)	
Drinkers	26 (16.9%)	31 (20.0%)	38 (24.5%)	
Exercise Habits(n/%)				0.070
Occasionally	100 (64.9%)	119 (77.3%)	118 (76.6%)	
Moderate intensity physical activity	40 (26.0%)	24 (15.6%)	23 (14.9%)	
Physical Activity	14 (9.1%)	11 (7.1%)	13 (8.4%)	

*RBC* red blood cell count*, WBC* white blood cell count, *TG* triglyceride, *TC* total cholesterol, *LDL-C* low-density lipoprotein cholesterol, *HDL-C* high density lipoprotein cholesterol, *ALT* alanine aminotransferase, *AST* aspartate transaminase, *BMI* body mass index, *SBP* systolic blood pressure, *DBP* diastolic blood pressure

*P* < 0.05

### Univariate analysis

Table 2 presents statistical insights into factors influencing LbaPWV and RbaPWV. Noteworthy correlations were observed in the univariate analysis.

**Table 2:** Univariate analysis of variables influencing LbaPWV and RbaPWV

	Statistics	RbaPWV (cm/s, β,95% CI, *P* value)	LbaPWV (cm/s, β,95% CI, *P* value)
SEX			
woman	252 (52.2%)	0	0
man	213 (45.8%)	−52.0 (−94.6, −9.3) 0.017	− 40.6 (− 86.4, 5.1) 0.083
Age (years, mean ± SD)	49.1 ± 9.5	14.5 (12.7, 16.4) < 0.0001	15.6 (13.7, 17.6) < 0.0001
RBC (*10^12/L, mean ± SD)	4.9 ± 0.5	39.5 (− 0.4, 79.4) 0.0532	28.7 (− 14.1, 71.5) 0.1890
WBC (*10^9/L, mean ± SD)	5.9 ± 1.5	−7.3 (− 21.2, 6.5) 0.3011	−6.5 (− 21.3, 8.3) 0.3893
Platelets (*10^9/L, mean ± SD)	191.6 ± 58.8	− 0.7 (− 1.0, − 0.3) 0.0003	− 0.7 (− 1.0, − 0.3) 0.0009
Height (cm, mean ± SD)	161.9 ± 8.4	− 3.2 (− 5.8, − 0.7) 0.0123	−4.0 (− 6.7, − 1.3) 0.0035
Weight (Kg, mean ± SD)	63.5 ± 11.4	1.6 (− 0.2, 3.5) 0.0875	1.4 (− 0.6, 3.4) 0.1690
BMI (kg/m2, mean ± SD)	24.1 ± 3.2	15.5 (8.9, 22.1) < 0.0001	16.1 (9.1, 23.2) < 0.0001
Waist (cm, mean ± SD)	83.1 ± 10.2	6.7 (4.6, 8.7) < 0.0001	6.8 (4.6, 9.0) < 0.0001
SBP (mmHg, mean ± SD)	124.5 ± 16.5	8.5 (7.4, 9.5) < 0.0001	9.0 (7.8, 10.1) < 0.0001
DBP (mmHg, mean ± SD)	75.1 ± 10.8	7.9 (6.1, 9.8) < 0.0001	8.2 (6.2, 10.2) < 0.0001
ALT (U/L, mean ± SD)	25.7 ± 21.3	1.0 (− 0.0, 2.0) 0.0586	0.9 (− 0.1, 2.0) 0.0836
AST (U/L, mean ± SD)	24.9 ± 14.4	1.7 (0.2, 3.2) 0.0255	1.8 (0.2, 3.3) 0.0296
TG (mmol/L, mean ± SD)	167.9 ± 161.4	0.2 (0.1, 0.3) 0.0033	0.2 (0.1, 0.3) 0.0059
TC (mmol/L, mean ± SD)	193.1 ± 36.3	1.0 (0.4, 1.6) 0.0010	1.0 (0.4, 1.6) 0.0018
HDL-C (mmol/L, mean ± SD)	67.2 ± 17.0	−3.0 (− 4.2, − 1.7) < 0.0001	−3.1 (− 4.5, − 1.8) < 0.0001
LDL-C (mmol/L, mean ± SD)	110.2 ± 27.2	1.1 (0.3, 1.9) 0.0054	1.1 (0.3, 2.0) 0.0081
Fasting blood glucose (mmol/, mean ± SD)	5.2 ± 1.0	56.3 (35.5, 77.0) < 0.0001	58.0 (35.7, 80.2) < 0.0001
Urea nitrogen (mmol/L, mean ± SD)	349.3 ± 92.2	0.2 (− 0.0, 0.4) 0.0897	0.2 (− 0.0, 0.4) 0.1126
Uric acid (umol/L, mean ± SD)	343.2 ± 93.3	0.3 (0.2, 0.3) < 0.001	0.2 (0.1, 0.3) < 0.001
Creatinine (umol/L,, mean ± SD)	67.3 ± 14.5	1.2 (0.5, 1.8) < 0.001	1.0 (0.3, 1.7) 0.004
Pepsinogen ratio (mean ± SD)	14.3 ± 6.1	− 7.6 (− 11.0, − 4.1) < 0.0001	− 8.6 (− 12.3, − 4.9) < 0.0001
Smoking(n/%)			
Nonsmokers	347 (74.8%)	0	0
Occasional smoking	25 (5.4%)	− 8.3 (− 104.0, 87.4) 0.8653	−25.0 (− 127.4, 77.3) 0.6318
Smokers	92 (19.8%)	11.7 (− 42.5, 65.9) 0.6715	8.4 (− 49.5, 66.4) 0.7758
Drinking(n/%)			
Nondrinkers	248 (53.4%)	0	0
Occasional alcohol consumption	121 (26.1%)	−6.8 (−58.1, 44.4) 0.7936	−24.8 (− 79.5, 30.0) 0.3759
Drinkers	95 (20.5%)	2.5 (−53.3, 58.2) 0.9304	−13.4 (−73.0, 46.2) 0.6594
Exercise Habits(n/%)			
Occasionally	337 (72.9%)	0	0
Moderate intensity physical activity	87 (18.8%)	16.8 (−38.6, 72.3) 0.5520	17.8 (− 41.5, 77.1) 0.5574
Physical Activity	38 (8.2%)	−31.5 (− 110.3, 47.4) 0.4348	−42.3 (−126.7, 42.1) 0.3260

*P* < 0.05

#### Gender

Specifically, RbaPWV was slower in males than in females (*P* = 0.017), whereas no significant difference was found for LbaPWV across genders.

#### Hematological markers

For instance, PGR was negatively correlated with both LbaPWV and RbaPWV (*P* < 0.05). Other hematological markers like RBC, WBC, and platelets didn’t show significant correlations.

#### Biochemical indices

AST, TG, TC, LDL-C, fasting blood glucose, uric acid, and creatinine positively correlated with LbaPWV/RbaPWV (*P* < 0.0001 for all). Conversely, HDL-C displayed a negative relationship. ALT and blood urea nitrogen were not significantly associated.

#### Lifestyle factors

Lifestyle variables like smoking status and alcohol consumption didn’t significantly impact LbaPWV/RbaPWV. Exercise habits, represented by categories such as occasional, moderate, and high-intensity activity, also failed to show any significant correlations.

### Association between PGR and baPWV: a multivariate analysis

To elucidate the relationship between PGR and baPWV, we employed multiple regression models. Two distinct adjustment models were used to confirm PGR’s independent association with RbaPWV and LbaPWV:

- **Model I**: Adjusted for gender and age
- **Model II**: Adjusted for all potential covariates identified in univariate analysis

As shown in Table 3, in the non-adjusted model, a unit increase in PGR was associated with a 7.6 cm/s decrease in RbaPWV and an 8.6 cm/s decrease in LbaPWV (*p* < 0.0001 for both). The negative association between PGR and RbaPWV/LbaPWV remained statistically significant in both Model I and Model II, affirming PGR’s independent negative relationship with RbaPWV and LbaPWV.

**Table 3:** Multivariate analysis of PGR’s association with RbaPWV/LbaPWV

	Non-adjusted (β,95% CI, *P* value)	Adjust I (β,95% CI, *P* value)	Adjust II (β,95% CI, *P* value)
RbaPWV (cm/s)			
PGR	−7.6 (−11.0, − 4.1) < 0.0001	− 4.7 (− 7.6, − 1.9) 0.0013	−3.1 (− 5.6, − 0.6) 0.0140
PGR Tertile			
Low	0	0	0
Middle	− 85.6 (− 136.6, −34.5) 0.0011	−55.9 (− 97.8, −14.0) 0.0093	−22.1 (− 58.6, 14.4) 0.2354
High	−129.0 (− 180.1, − 77.9) < 0.0001	− 80.1 (− 122.6, − 37.6) 0.0002	−52.9 (− 90.6, − 15.2) 0.0062
LbaPWV (cm/s)			
PGR	−8.6 (− 12.3, − 4.9) < 0.0001	−5.4 (− 8.4, − 2.3) 0.0006	−3.9 (− 6.5, − 1.2) 0.0051
PGR Tertile			
Low	0	0	0
Middle	−91.5 (− 146.1, − 36.8) 0.0011	− 58.7 (− 103.5, − 13.9) 0.0105	− 23.3 (− 62.6, 16.0) 0.2463
High	− 137.9 (− 192.6, − 83.3) < 0.0001	− 83.3 (− 128.7, − 37.8) 0.0004	−55.7 (− 96.4, − 15.0) 0.0076

*P* < 0.05

### Nonlinear relationship analyses between PGR and baPWV

**Fig. 1:**
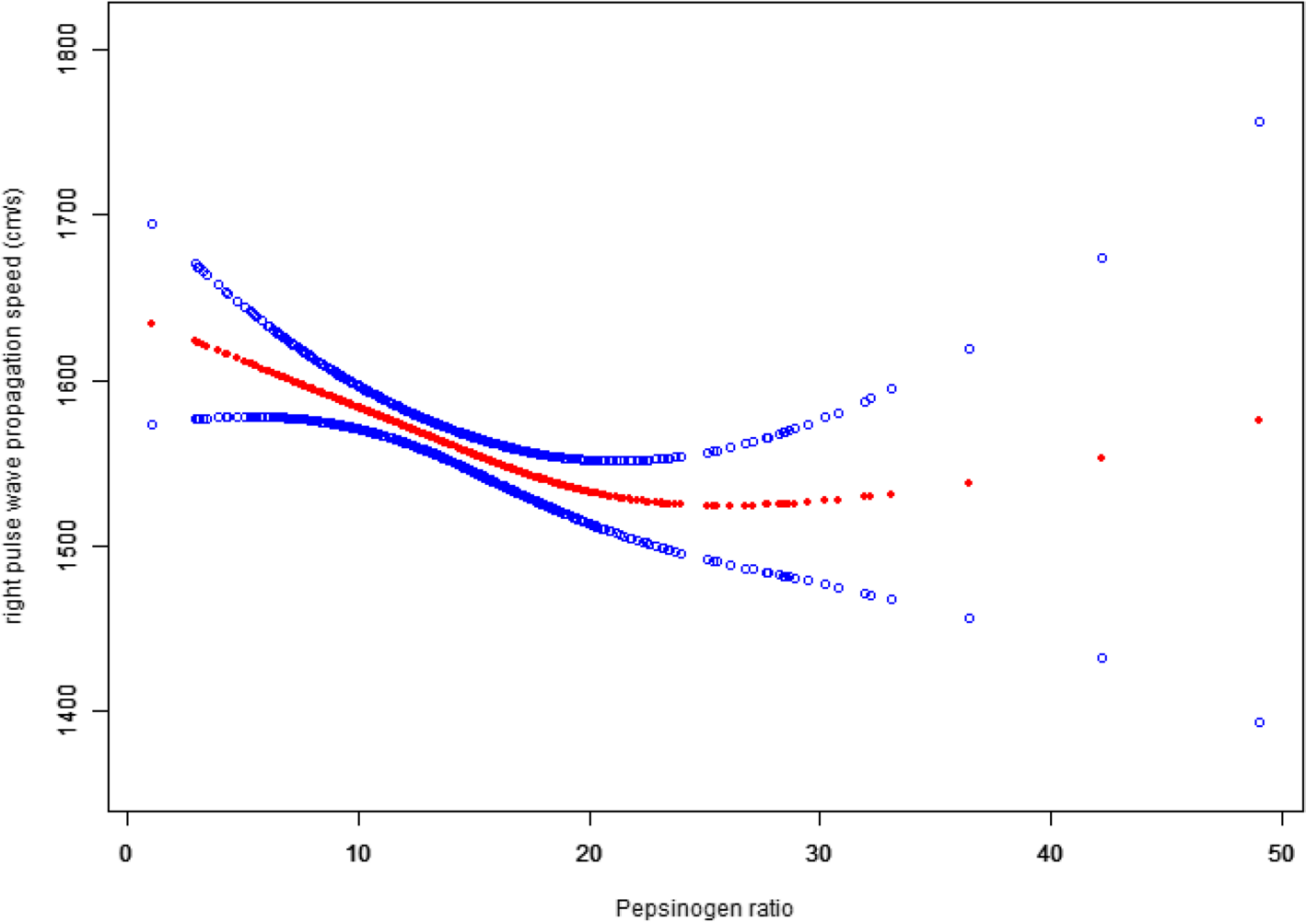
Correlation curve between PGR and RbaPWV

**Fig. 2:**
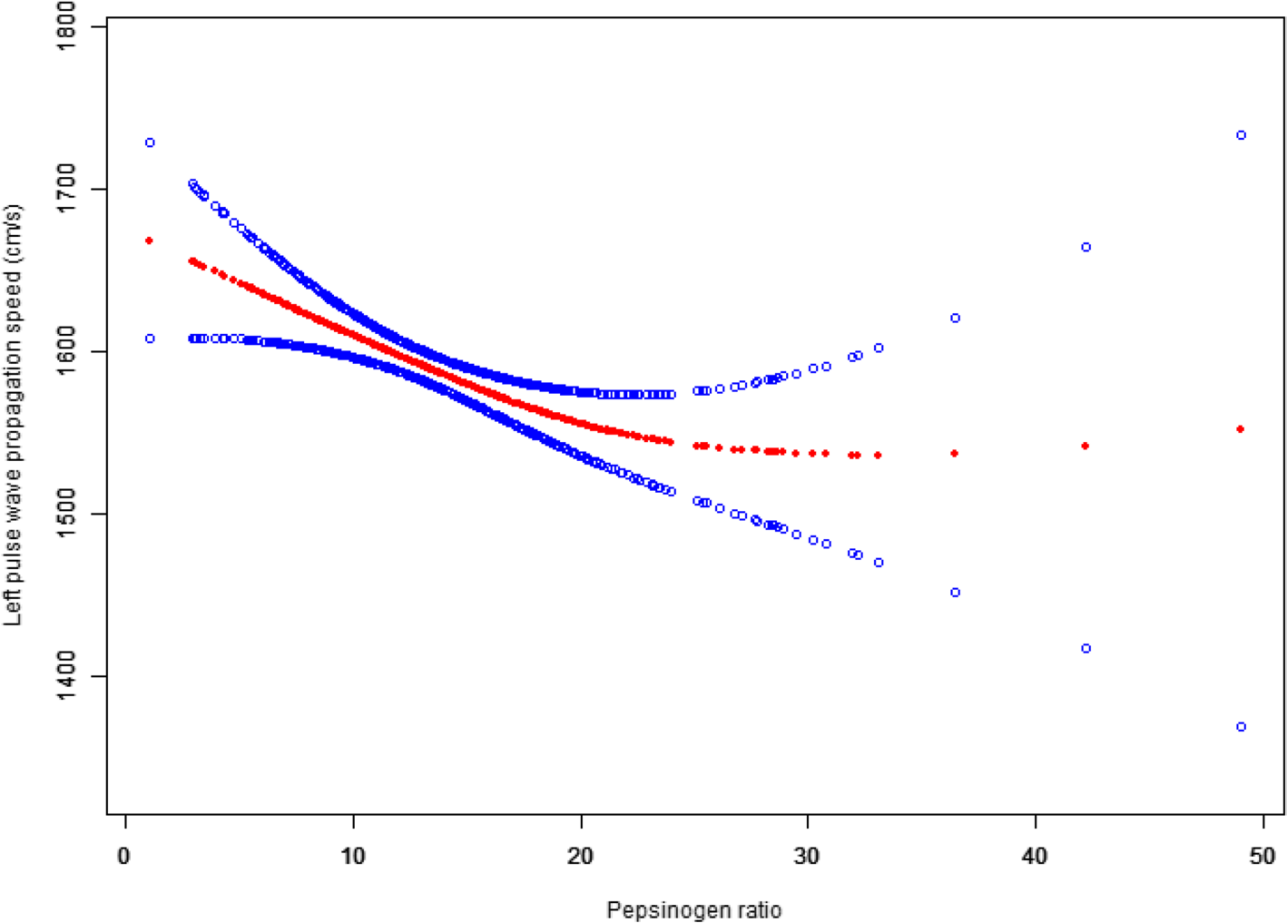
Correlation curve between PGR and LbaPWV

To investigate the linearity of the relationship between PGR and both RbaPWV and LbaPWV, smooth curve fitting was employed. Upon adjusting for all confounding variables, we observed a nonlinear association between PGR with both RbaPWV and LbaPWV, as visualized in Figs. 1 and 2. To identify the inflection points in these nonlinear relationships, segmented regression models were applied. The identified inflection points for PGR against RbaPWV and LbaPWV were 22.5 and 22.4, respectively. These findings are summarized in Table 4.

**Table 4:** Nonlinear concentration-effect relationship of PGR with baPWV

Outcome:	RbaPWV (cm/s, β,95% CI, *P* value)	LbaPWV (cm/s, β,95% CI, *P* value)
Knockout point (K)	22.5	22.3
< K-segment effect value	−6.3 (− 9.6, − 3.0) < 0.001	− 7.0 (− 10.6, − 3.4) < 0.001
> K-segment effect value	5.6 (−1.8, 12.9) 0.141	4.4 (− 3.4, 12.2) 0.273

Exposure variable: PGR

*P* < 0.05

For PGR values below 22.5, each unit increase in PGR was corresponded with a 6.3 cm/s decrease in RbaPWV (*p* < 0.001). Conversely, for PGR values exceeding 22.5, each unit increase resulted in a statistically insignificant 5.6 cm/s increase in RbaPWV (*p* = 0.141). Similarly, when PGR values below 22.3, each unit increase was linked to a 7.0 cm/s decrease in LbaPWV (p < 0.001). For PGR values above 22.3, however, each unit increase led to an insignificant 4.4 cm/s increase in RbaPWV (*p* = 0.273).

## Discussion

The study aimed to investigate the correlation between PGR and baPWV. As we predicted, both RbaPWV and LbaPWV were significantly correlated with patients’ PGR levels. Negative correlations between PGR and RbaPWV/LbaPWV were observed in the unadjusted model. Furthermore, this negative correlation persisted even after adjusting for gender, age, and other potential confounding factors.

Smooth curve fitting revealed a nonlinear relationship between PGR and both RbaPWV and LbaPWV. We identified inflection points at 22.5 and 22.3, respectively. Below these inflection points, each unit increase in PGR was associated with a 6.3 cm/s decrease in RbaPWV and a 7.0 cm/s decrease in LbaPWV. Given that PGI primarily originates from gastric chief cells, while PGII is derived from all gastric glands as well as duodenal mucosa [15, 16], a decline in PGR levels occurs in the context of chronic gastric mucosal atrophy.

A study conducted in 2012 investigated the risk of myocardial infarction, stroke, and all-cause mortality, concluding that chronic atrophic gastritis —defined by PGI levels below 70 ng/mL and a PGI/PGII ratio under 3—was not a significant risk factor for cardiovascular disease or mortality [17]. Conversely, a 2021 study contrasted PGI, PGII, and PGR levels between patients with atherosclerotic cardiovascular disease (ASCVD) and a healthy control group. This research indicated that low PGR levels increased risk factor for ASCVD, whereas high PGR levels were inversely correlated with ASCVD [9]. The divergence in findings could be attributed to the initial focus of the 2012 study on chronic atrophic gastritis (PGI < 70 ng/mL and PGI/PGII < 3) as a baseline for subsequent analyses. In actuality, the cardiovascular risks among the study subjects might have escalated prior to any notable change in PGI and PGII levels, which are triggered by damage to gastric chief cells. Our investigation determined that when PGR fell below 22.3, a linear negative correlation was established between PGR and RbaPWV; similarly, when PGR was under 22.5, a linear negative correlation was established between PGR and LbaPWV. These results, aligning with the 2021 study, affirm that PGR levels and atherosclerosis are interrelated.

This supports the notion that arterial stiffness is present prior to the onset of gastric mucosal atrophy, which is defined by a PGR greater than 3. Previous research has demonstrated that serum PGI levels decrease as one ages, whereas PGII levels generally increase in individuals under the age of 60 and decline in those over 60 [18]. Consequently, PGR tends to rise before the age of 60 and diminish thereafter. A study published in 2017 further indicated that PGR is influenced by additional variables such as *Helicobacter pylori* infection, renal function, and fasting blood glucose levels [19]. Age, renal impairment, and elevated fasting glucose are already recognized as risk factors for atherosclerosis. Henceore, these factors may act as mediators linking a decline in PGR with the onset of atherosclerosis. Notably, our research discovered that even after accounting for these and other confounding variables like gender and creatinine levels, the inverse correlation between PGR and baPWV remained significant.

Elevated intravascular pressure and increased shear stress resulting from atherosclerosis contribute to endothelial dysfunction and stimulate the production and deposition of collagen in the arterial wall, factors closely associated with atherosclerosis itself [20]. PWV is the most widely used metric for gauging arterial stiffness. Existing literature not only identifies a link between PWV and coronary atherosclerosis but also establishes a positive correlation between PWV and the severity of coronary artery disease [21, 22]. Furthermore, studies indicate that PWV correlates not just with cerebral and cerebellar vascular diseases, but also with the thickening of the carotid intima-media and the formation of carotid plaques [23–25]. These findings suggest that PWV is positively correlated with the progression of atherosclerosis [26]. Contrary to these trends, our research identified an inverse relationship between PGR levels and atherosclerosis. This implies that manipulating PGR levels could be a viable strategy for mitigating the progression of atherosclerosis in patients.

Our research established that PGR, a crucial marker for gastrointestinal function, also has a correlated with peripheral vascular stiffness. This observation could offer novel perspectives for the future prevention and treatment of arterial atherosclerosis. Nonetheless, the mechanisms underlying the decline in PGR levels and its subsequent influence on arterial stiffness require further investigation.

However, the study is not without limitations. Conducted at a single center and primarily involving participants from the indigenous population in southern China, he results may not be universally applicable to diverse populations in other regions. Furthermore, the study’s cross-sectional design allows for establishment for association between PGR and RbaPWV/LbaPWV but does not provide evidence for causal relationships. Consequently, future research should aim for multi-center studies to validate to the generalizability and reliability of these findings across varied populations and to further investigate the causal links between PGR and arterial stiffness.

## Conclusion

Our findings establish a linear negative correlation between reduced PGR levels and elevated baPWV. Conversely, the relationship between elevated PGR levels and RbaPWV/LbaPWV exhibits a nonlinear pattern. The observed correlations between PGR and RbaPWV/LbaPWV indicate that regulation of PGR levels could serve as a potential strategy for mitigating atherosclerosis in patients.

### Supplementary Information

**Additional file 1: Supplementary Figure 1.** Correlation curve between HDL-C and RbaPWV. Correlation between HDL-C and RBaPWV is shown in Supplementary Figure 1. where the red line represents a smooth curve fitting of the correlation between HDL-C and RBaPWV, and the distance between the two blue dashed lines represents their 95% confidence intervals.

**Additional file 2: Supplementary Figure 2.** Correlation curve between HDL-C and LbaPWV. Correlation between HDL-C and LBaPWV is shown in Supplementary Figure 2. where the red line represents a smooth curve fitting of the correlation between HDL-C and LBaPWV, and the distance between the two blue dashed lines represents their 95% confidence intervals.

## Data Availability

The data presented in this study are available on request from the corresponding authors.

## Abbreviations

PGR: Pepsinogen ratio
baPWV: Rachial-ankle pulse wave velocity
TG: Triglycerides
TC: Total cholesterol
HDL-C: High-density lipoprotein cholesterol
LDL-C: Low-density lipoprotein cholesterol
ALT: Alanine aminotransferase
AST: Aspartate transaminase
SBP: Systolic blood pressure
DBP: Diastolic blood pressure
BMI: Body mass index
RBC: Red blood cell count
WBC: White blood cell count
MET: Metabolic equivalent
IPAQ: International physical activity questionnaire
ASCVD: Atherosclerotic cardiovascular disease
