# Supplementary figures and images for: Pepsinogen ratio and brachial-ankle pulse wave velocity: a cross-sectional study on their interrelationship in atherosclerosis
Source: BMC Cardiovasc Disord. 2023 Nov 21;23:572. doi: 10.1186/s12872-023-03618-9 (PMC10662786; doi:10.1186/s12872-023-03618-9)

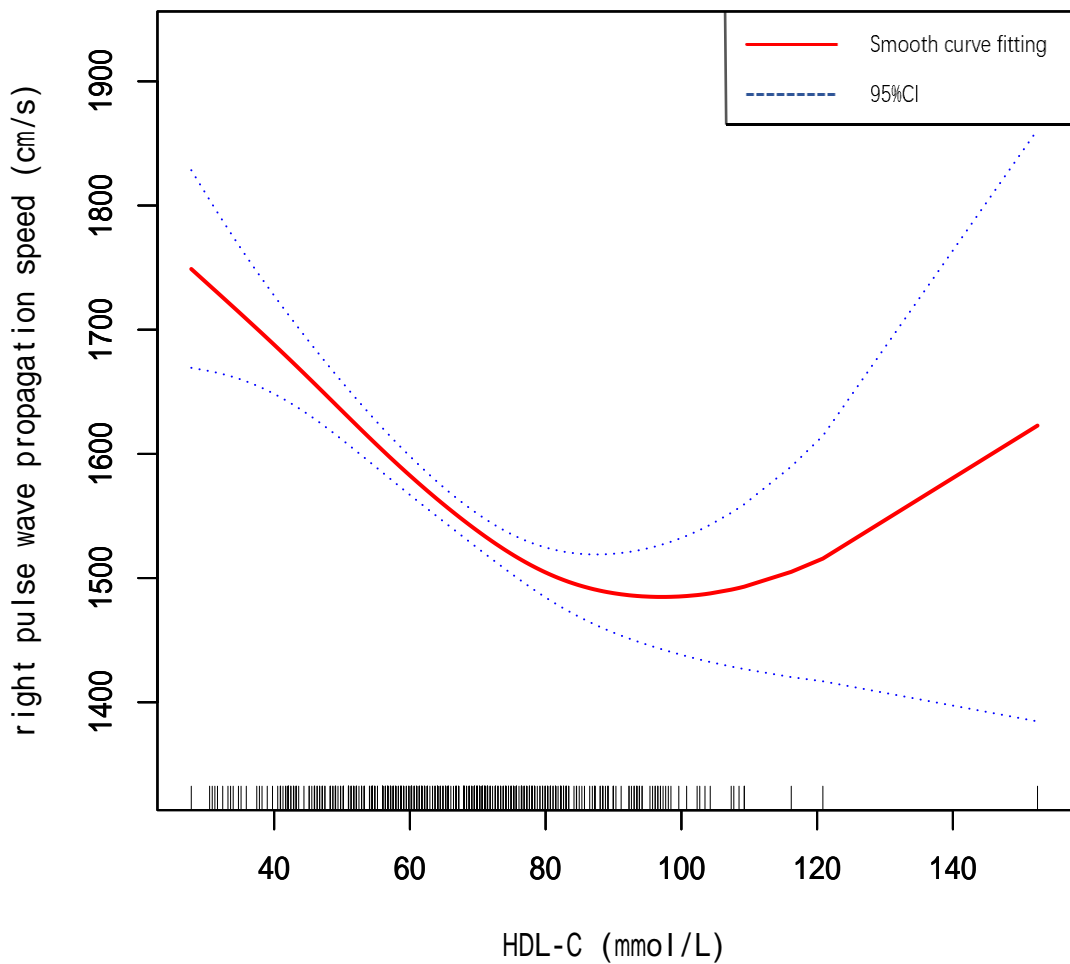

Supplement: Supplementary file 1 — Additional file 1: Supplementary Figure 1. Correlation curve between HDL-C and RbaPWV. Correlation between HDL-C and RBaPWV is shown in Supplementary Figure 1. where the red line represents a smooth curve fitting of the correlation between HDL-C and RBaPWV, and the distance between the two blue dashed lines represents their 95% confidence intervals. [file 12872_2023_3618_MOESM1_ESM.pdf]

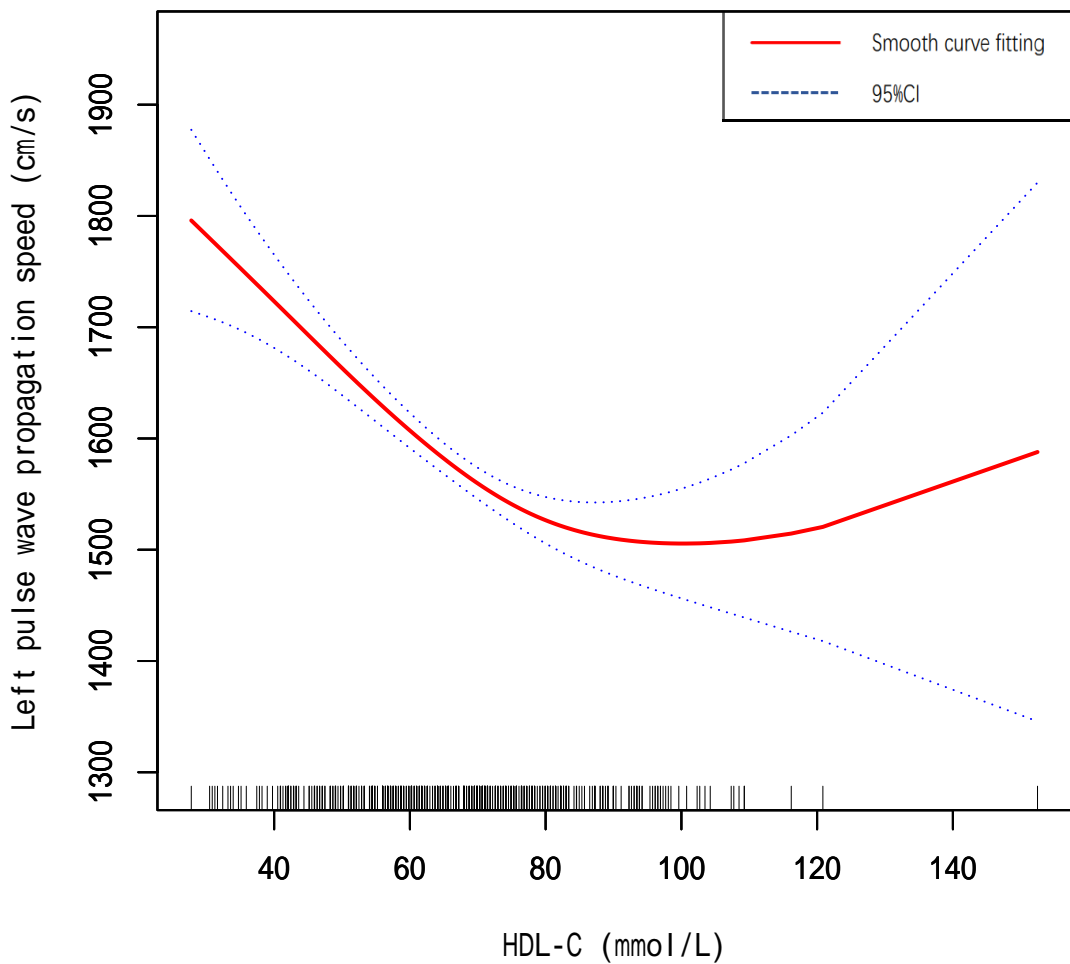

Supplement: Supplementary file 2 — Additional file 2: Supplementary Figure 2. Correlation curve between HDL-C and LbaPWV. Correlation between HDL-C and LBaPWV is shown in Supplementary Figure 2. where the red line represents a smooth curve fitting of the correlation between HDL-C and LBaPWV, and the distance between the two blue dashed lines represents their 95% confidence intervals. [file 12872_2023_3618_MOESM2_ESM.pdf]
